# Coffee polyphenols exert hypocholesterolemic effects in zebrafish fed a high-cholesterol diet

**DOI:** 10.1186/1743-7075-10-61

**Published:** 2013-10-03

**Authors:** Shinichi Meguro, Takahiro Hasumura, Tadashi Hase

**Affiliations:** 1 Biological Science Research, Kao Corporation, 2606 Akabane, Ichikai-machi, Haga-gun, Tochigi 321-3497, Japan

**Keywords:** Chlorogenic acids, Cholesterol diet, Coffee polyphenols, Cytochrome P450 7A1, 3-hydroxy-3- methylglutaryl-coenzyme A reductase, Hypercholesterolemia, Microsomal triglyceride transfer protein, Vascular lipid accumulation, Zebrafish

## Abstract

**Background:**

Hypercholesterolemia is an important risk factor for the development of coronary artery disease. Some dietary polyphenols, such as coffee polyphenols (CPPs), reduce cholesterol levels. The mechanism of this cholesterol-lowering effect is not fully understood, although 5-CQA, a major component of CPPs, reportedly inhibits cholesterol biosynthesis. Here, we investigated the mechanism of the cholesterol-lowering effect of CPPs on the basis of cholesterol metabolism–related gene expression in the liver. We also examined the effects of CPPs on vascular lipid accumulation in zebrafish with high cholesterol diet–induced hypercholesterolemia.

**Methods:**

Over 14 weeks, adult zebrafish were fed a control diet, a high-cholesterol diet, or the latter diet supplemented with CPPs. To measure the extent of vascular lipid accumulation, for 10 days larval zebrafish (which are optically transparent) were fed these same diets with the addition of a fluorescent cholesteryl ester.

**Results:**

In adult zebrafish, addition of CPPs to a high-cholesterol diet significantly suppressed the increase in plasma and liver cholesterol levels seen when fish ingested the same diet lacking CPPs. Transcription levels of the liver genes *hmgcra* (encoding 3-hydroxy-3-methylglutaryl-coenzyme A reductase A, a rate-limiting enzyme in cholesterol biosynthesis) and *mtp* (encoding microsomal triglyceride transfer protein, a lipid transfer protein required for assembly and secretion of lipoproteins) were significantly lower in fish fed the CPP-containing diet than in fish fed the unsupplemented high-cholesterol diet. In contrast, the expression level of the liver gene *cyp7a1a* (encoding the cytochrome P450 polypeptide 1a of subfamily A of family 7, a rate-limiting enzyme for bile acid biosynthesis) increased significantly upon consumption of the CPP-containing diet. In larval fish, accumulation of fluorescently labeled cholesterol in the caudal artery was greatly reduced on the CPP-containing diet.

**Conclusions:**

CPP ingestion suppressed cholesterol accumulation in the plasma, liver, and vascular system of zebrafish. Downregulation of cholesterol and lipoprotein synthesis and upregulation of bile acid synthesis in the liver may be the fundamental underlying mechanisms by which CPPs exert their hypocholesterolemic effects. CPP intake may help prevent and manage hypercholesterolemia in humans, and further investigations along these lines using a variety of CPP dose rates are warranted.

## Background

Cardiovascular disease is the leading cause of morbidity and mortality worldwide. Hypercholesterolemia triggers atherosclerosis and is a major risk factor for the development of cardiovascular disease. Several epidemiological studies have clearly shown that increased levels of plasma cholesterol—particularly low-density lipoprotein (LDL) cholesterol—are associated with the development of cardiovascular disease [1,2]. Importantly, hypercholesterolemia is a modifiable risk factor. Lifestyle changes and ingestion of various dietary compounds—such as green tea, plant sterols, and soy proteins—can reduce hypercholesterolemia [3].

Worldwide, coffee is one of the most widely consumed beverages, and it is an extremely rich source of biologically active polyphenols [4]. Coffee polyphenol (CPP) preparations are rich in caffeoylquinic acids (CQAs), feruloylquinic acids (FQAs), and dicaffeoylquinic acids (diCQAs). The physiological activities of CPPs include dose-dependent reduction in blood pressure, improvement of endothelial function, and suppression of diet-induced body fat accumulation and postprandial hyperglycemia [5-10]. Recently, the reduction of cholesterol levels by CPPs and 5-CQA (chlorogenic acid) has been reported [9,11-14]. However, little has been reported about the mechanism of action of these substances: it has been reported only that 5-CQA inhibits cholesterol biosynthesis [11,14]. None of the possible effects of CPPs on vascular lipid accumulation—an important early step in atherogenesis—has been studied.

Zebrafish (*Danio rerio*) organs and tissues are similar to those of humans in both structure and function. Consequently, the zebrafish is increasingly being used as a model of human disease because (a) the fish is readily amenable to genetic manipulation; (b) it breeds readily in captivity; and, (c) experimental colonies can be inexpensively maintained [15]. In addition, several studies have found that the zebrafish is an excellent model of vertebrate lipid metabolism [16,17]. Stoletov et al. [18] showed that zebrafish fed a high-cholesterol diet developed hypercholesterolemia, exhibited a remarkable capacity to oxidize lipoproteins and accumulated vascular lipids. They concluded that the zebrafish was a suitable model in which to study the pathological events important in early-stage atherogenesis.

Here, we investigated the effects of CPP ingestion by zebrafish rendered hypercholesterolemic by consumption of a high-cholesterol diet. We measured plasma and liver cholesterol levels, the expression levels of liver genes involved in cholesterol metabolism, and the extent of vascular lipid accumulation. We compared data from test and control fish cohorts.

## Methods

### Preparation of CPP

CPPs were prepared by hot-water extraction from roasted coffee beans, and CPP composition was determined by HPLC analysis of the extract [9]. The total polyphenol content of the CPP preparation was 77.1% (w/w). Individual polyphenol compositions were (all w/w): 7.7% 3-CQA; 15.5% 4-CQA; 31.9% 5-CQA; 7.6% 3- FQA; 5.6% 4-FQA; 8.5% 5-FQA; 9.0% 3,4-diCQA; 8.9% 3,5-diCQA; and 5.3% 4,5-diCQA. The CPP powder contained no caffeine.

### Animals

Adult zebrafish were purchased from a local pet supplier (Meito Suien Co., Ltd., Remix, Nagoya, Japan). All fish were raised and maintained on a 14:10-h light:dark cycle at 28°C, and water quality was maintained as described in *The Zebrafish Book*[19]. All fish were handled in strict accordance with the Guidelines of the Animal Care Committee of Kao Corporation, *The Zebrafish Book*, and the *Guide for the Care and Use of Laboratory Animals* (8th edition) [20].

### Diets

The control diet contained 75% (w/w) standard zebrafish chow (Otohime B2; Marubeni Nisshin Feed Co. Ltd., Tokyo, Japan) and 25% (w/w) gluten (Wako Pure Chemical Industries, Ltd., Osaka, Japan). The high-cholesterol diet was the control diet with the addition of 4% (w/w) cholesterol (Wako Pure Chemical Industries). The “CPP diet” was the control diet with the addition of both 4% (w/w) cholesterol and 5% (w/w) CPPs. To measure the cholesterol content of the Otohime B2 chow, lipids were extracted by using the conventional Folch method [21]. The cholesterol content of the control diet before the addition of cholesterol was 0.4% (w/w).

### Experimental design

*Experiment 1*: Female adult zebrafish 6 months post-fertilization were weighed under anesthesia with 0.005% (w/v) tricaine (Sigma-Aldrich, MO, USA) and allocated to three groups of 7 or 8 fish with similar body weights. Each group was placed in a 1.7-L tank. The three groups were fed the control, the high-cholesterol, or the CPP diet twice daily (20 mg of food/day per fish) over 14 weeks. During feeding, water inflow to the tanks was paused for 45 min and the fish were allowed to consume their diet for 30 min. On the last day of the experiment, all fish were euthanized, body weights were measured, and blood samples were taken from each caudal artery (into heparinized glass capillaries) and dissected livers. Samples from each liver were stored in duplicate for analysis of lipid levels and mRNA expression patterns.

*Experiment 2*: Twice daily for 30 min for 10 days, zebrafish larvae (collected 5 days post-fertilization) were fed either the control, the high-cholesterol, or the CPP diet supplemented with 0.002% (w/w) cholesteryl BODIPY 576/589 C11 (Life Technology, CA, USA). The larvae were maintained in egg water (water containing 0.6 g NaCl/L) at 28°C under light-shielded conditions. The egg water was changed after each feeding. On the final day of the experiment, 10 randomly selected larvae from each group were collected and anesthetized by being placed in a small drop of 0.005% (w/v) tricaine; they were then placed onto a glass slide and immediately transferred into the light-shielded chamber of a fluorescence microscope (BZ-9000; Keyence Corporation, Osaka, Japan), and a fluorescence image of the caudal artery (1000 μm caudally from just above the anus) was photographed. The specifications of the BZ-9000 were as follows: light source, ultra-high-pressure mercury lamp (120 W); fluorescence filter, BZ filter TRITC (excitation wavelength 540 nm, absorption wavelength 605 nm); objective, Nikon CFI 60 series (×10); and camera, 2/3 inch, 1.5 million pixel monochrome CCD. To ensure sufficient sensitivity of the comparisons of fluorescence among larvae, each larva was placed on the stage for no more than 1 min. The mean fluorescence intensity of the caudal arteries of each group was quantified by specifying the range of fluorescence of the caudal artery with the aid of inbuilt BZ-II Analyzer software (Keyence Corporation).

### Analysis of plasma and hepatic lipid levels

Plasma was obtained by centrifugation of blood samples at 1500*g* for 5 min at 4°C. Liver lipids were extracted by using the conventional Folch method [21]. Cholesterol levels in plasma and liver were enzymatically determined with Cholesterol E-Test kits (Wako Pure Chemical Industries, Ltd).

### RNA extraction and quantitative real-time PCR

Total RNA was extracted from the liver samples of the fishes in Experiment 1 by using RNeasy Lipid Tissue Mini-Kits (Qiagen K.K., Tokyo, Japan) and was transcribed into cDNA by using a High Capacity RNA-to-cDNA (Applied Biosystems, CA, USA). Quantitative real-time PCR was performed on an ABI PRISM platform (Applied Biosystems); analysis of cDNA samples employed the TaqMan Fast Universal PCR Master Mix. The TaqMan assays explored the expression levels of the following genes: *eef1a1l1* (eukaryotic translation elongation factor 1 alpha 1, like 1; Dr03432748_m1); *hmgcra* (3-hydroxy-3-methylglutaryl-coenzyme A reductase A; Dr03428716_m1); *mtp* (microsomal triglyceride transfer protein; Dr03133293_m1); and *cyp7a1a* (polypeptide 1a of subfamily A of family 7 of cytochrome P450; Dr03177268_s1). Each baseline and threshold level was manually set in line with the manufacturer’s instructions. Relative mRNA expression levels were determined by using the expression level of *eef1a1l1* as an internal standard.

### Statistical analysis

All data are shown as means ± SE (standard error). The significance of observed differences was evaluated via analysis of variance followed by application of Fisher’s partial least-squares difference multiple comparison. A difference was considered to be significant if the comparative *P*-value was <0.05. Statistical calculations were performed with the aid of Stat-View for Windows (version 5.0; SAS Institute Inc., NC, USA).

## Results

### Food intake and body weight of adult zebrafish

**Figure 1:**
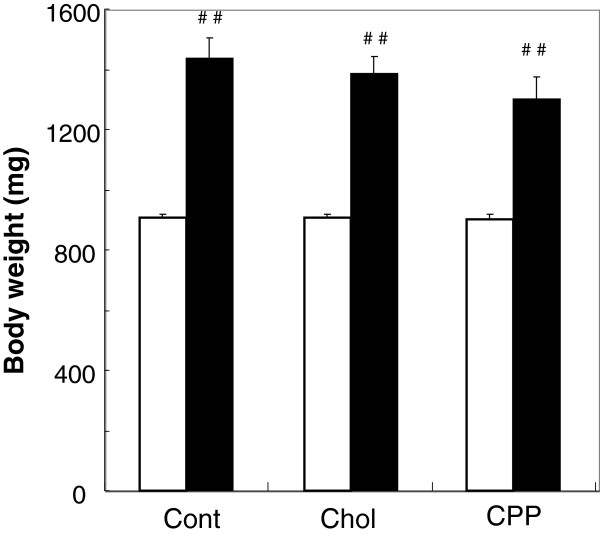
**Body weights of zebrafish before, and at the conclusion of, Experiment 1.** For 14 weeks, female adult zebrafish (6 months post-fertilization) were fed a control diet, a high-cholesterol diet, or a CPP diet (20 mg food/day per fish; n = 15 or 16 in each group). White and black bars show initial and final body weights, respectively. Values are means ± SE. ^##^*P* < 0.01 compared with initial weight. Cont: control diet; Chol: high-cholesterol diet; CPP: CPP diet (high-cholesterol diet supplemented with CPP).

Food intake in Experiment 1 was estimated by daily observation. During the experimental period, all fish ate up each diet completely (20 mg of food/day per fish). Body weight increased significantly (*P* < 0.01) in each group over the experimental period, and the among-group differences were not significant (Figure 1).

### Plasma and liver cholesterol levels in adult zebrafish

**Figure 2:**
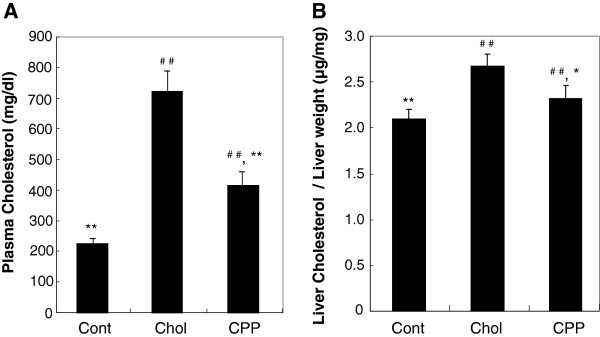
**Effects of CPPs on plasma and liver cholesterol levels.** Plasma **(A)** and liver **(B)** cholesterol levels are shown in fish fed a control diet, a high-cholesterol diet, or a CPP diet (n = 15 or 16 in each group). Values are means ± SE. ^##^*P* < 0.01 vs. fish fed a control diet; *P < 0.05; ^**^P < 0.01 vs. fish fed a high-cholesterol diet.

We examined plasma and liver cholesterol levels at the end of Experiment 1. The plasma cholesterol level was significantly higher (*P* < 0.01) in fish fed the high-cholesterol diet (722.3 ± 75.5 mg/dl) than in fish fed the control diet (225.0 ± 17.9 mg/dl), but this rise was significantly suppressed (*P* < 0.01) in fish fed the CPP diet (416.0 ± 41.7 mg/dl) (Figure 2A). The liver cholesterol level was significantly higher (*P* < 0.01) in fish fed the high-cholesterol diet (2.68 ± 0.13 μg/mg liver weight) than in fish fed the control diet (2.10 ± 0.11 μg/mg liver weight). This rise was significantly suppressed (*P* < 0.05) in fish fed the CPP diet (2.32 ± 0.15 μg/mg liver weight) (Figure 2B).

### Expression of mRNAs in the liver of adult zebrafish

**Figure 3:**
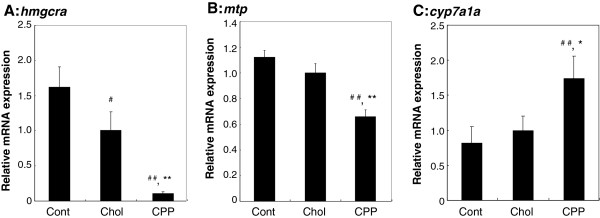
**Effect of CPPs on levels of expression of genes involved in cholesterol metabolism in the liver.** Levels of mRNA transcribed from the genes *hmgcra***(A)**, *mtp***(B)**, and *cyp7a1a***(C)** in the livers of zebrafish fed a control diet, a high-cholesterol diet, or a CPP diet (n = 15 or 16 in each group) are shown. mRNA levels were normalized against that transcribed from *eef1a1l1*. Values are means ± SE. ^#^*P* < 0.05; ^##^*P* < 0.01 vs. fish fed a control diet; *P < 0.05; **P < 0.01 vs. fish fed a high-cholesterol diet.

In an effort to define the mechanism by which CPPs exerted their anti-hypercholesterolemic effects, we measured the mRNA expression levels of genes associated with cholesterol metabolism in the liver. The levels of mRNA transcribed from *hmgcra* and *mtp* were significantly lower (*P* < 0.01), and that from *cyp7a1a* significantly higher (*P* < 0.05), in fish fed the CPP diet than in fish fed the high-cholesterol diet (Figure 3A, B, and C).

### Vascular cholesterol accumulation in larval zebrafish

**Figure 4:**
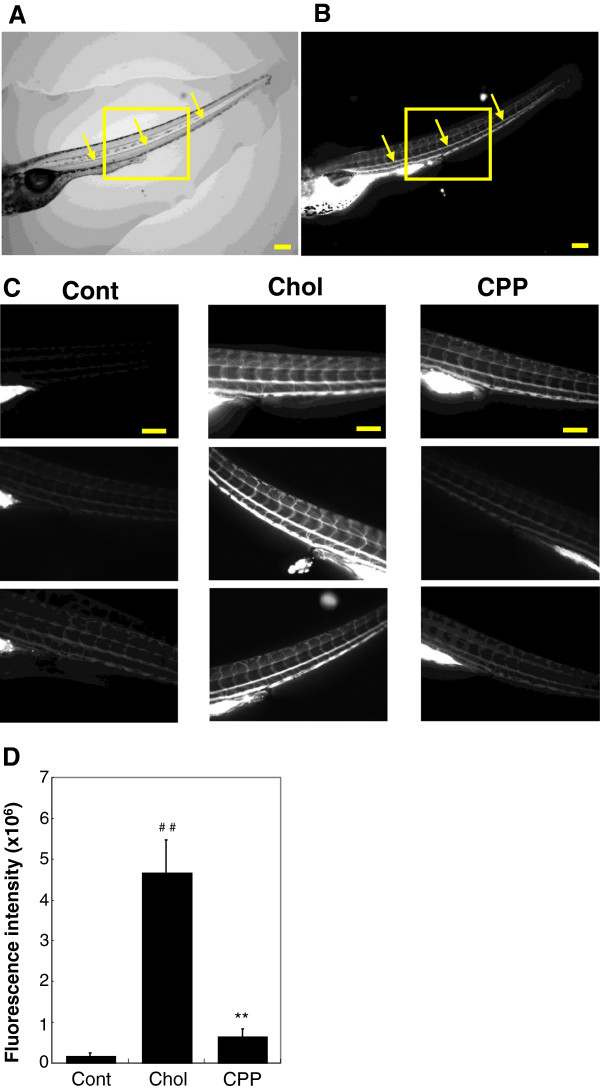
**Effect of CPPs on vascular cholesterol accumulation in larval zebrafish.** Larval zebrafish at 5 days post-fertilization were fed a control diet, a high-cholesterol diet, or a CPP diet; all diets additionally contained 0.002% (w/w) of the fluorescent cholesteryl ester BODIPY 576/589 C11. Diets were administered over 10 days. **A**, **B**: Optical **(A)** and fluorescent **(B)** images taken of zebrafish larvae at the end of the experiment. Arrows in these images identify the caudal artery. Scale bars: 200 μm. **C**: Fluorescence images of zebrafish larvae from the trunk to the tail, including the caudal artery (outlined by rectangles in Figure 4A and B), taken after 10 days of consumption of a diet supplemented with fluorescent cholesterol. Scale bars: 200 μm. **D**: Fluorescence intensities of the caudal arteries of fish from the three dietary groups (n = 10). Values are means ± SE. ^##^*P* < 0.01 vs. fish fed a control diet; ^**^P < 0.01 vs. fish fed a high-cholesterol diet.

We explored the effect of CPP dietary supplementation on the extent of vascular cholesterol accumulation in the caudal arteries of optically transparent zebrafish larvae. Optical and fluorescence images are shown (Figure 4A and B, respectively). Cholesterol tagged with a fluorescent marker accumulated in the caudal artery (arrows in Figure 4B). Figure 4C shows fluorescence images from the trunk to the tail (including the caudal artery) of fish from each dietary group. The caudal artery fluorescence level of cholesterol tagged with a fluorescent marker was significantly greater (*P* < 0.01) in fish fed the high-cholesterol diet than in fish fed the control diet, but this increase was tempered significantly (*P* < 0.01) when the high-cholesterol diet was supplemented with CPPs (Figure 4D).

## Discussion

We explored whether supplementation of a high-cholesterol diet with CPPs would mitigate the hypocholesterolemic effect of the former diet in zebrafish. Addition of CPPs to the high-cholesterol diet suppressed the increase in plasma cholesterol levels induced by the unsupplemented diet. Cho et al. [11] and Wan et al. [12] reported that addition of 5-CQA, a major component of CPPs, to high-fat or high-cholesterol diets suppressed the increases in plasma cholesterol levels normally seen when such unsupplemented diets were fed to rodents. In our study, 5-CQA constituted 31.9% (w/w) of the added CPPs; 5-CQA was thus the principal polyphenol present. Our results suggest that dietary CPPs lower plasma cholesterol levels partly because CPPs have high levels of 5-CQA. In addition, we found that CPP supplementation suppressed the increase in hepatic cholesterol level triggered by ingestion of a high-cholesterol diet. Murase et al. [9] also found that CPPs suppressed the accumulation of hepatic cholesterol in mice fed a high-fat diet. Together, the data suggest that CPPs exert a hypocholesterolemic effect by influencing cholesterol metabolism in the liver. CPPs ingested in food or drink (coffee) may be useful in prevention and management of hypercholesterolemia.

Hepatic mRNA expression analysis revealed that CPP supplementation decreased transcription of *hmgcra* and *mtp* but elevated the transcriptional level of *cyp7a1a*. In the liver, the protein encoded by *hmgcra* is a rate-limiting enzyme in cholesterol biosynthesis and the target of statins, which are popular cholesterol-lowering drugs [22,23]. Thus, the observed decrease in expression of *hmgcra* may be one mechanism by which CPPs exert their hypocholesterolemic effects. On the other hand, mtp encodes an enzyme that is a lipid transfer protein required for the assembly and secretion of lipoproteins [24]. The protein encoded by *cyp7a1a* is rate-limiting enzyme for cholesterol catabolism; the enzyme converts cholesterol to bile acids [25]. Indeed, hypercholesterolemia induced by a high-cholesterol diet is not evident in liver-specific conditional *Mttp* knockout mice [26]. Transgenic mice overexpressing *Cyp7a1* in the liver exhibit increased levels of hepatic cholesterol catabolism, a rise in the bile acid pool, and a decreased concentration of plasma cholesterol [27]. Therefore, we speculate that downregulation of cholesterol and lipoprotein synthesis, with concomitant upregulation of bile acid synthesis, underlies the hypocholesterolemic effects of CPP ingestion.

Cho et al. [11], cited above, showed that the hypocholesterolemic effects of 5-CQA could be attributed to a decrease in HMGCR enzymatic activity in the rodent liver. Such downregulation of activity, attributable to a fall in the level of mRNA transcription from *Hmgcr*, has been suggested to be the fundamental mechanism whereby the dietary polyphenols epigallocatechin-3-gallate, resveratrol, curcumin, and hesperetin exert hypocholesterolemic effects [28-33]. Although the structure–activity relationships of these polyphenols remain poorly understood, the accumulated data encourage us to speculate that polyphenolic compounds regulate hepatic *Hmgcr* expression and thus HMGCR levels. If so, the downregulation of hepatic *hmgcra* expression upon CPP dietary supplementation may be attributable not only to the action of 5-CQA, but also to the activities of other CPPs.

The caudal artery increase in accumulation of a fluorescent form of cholesterol in the fish fed a high-cholesterol diet was inhibited by concomitant ingestion of CPPs. Fang et al. [34] found that when larval zebrafish were fed a high-cholesterol diet for only 2 weeks the levels of certain oxidized cholesteryl esters, identical to those present in minimally oxidized LDL of humans and murine atherosclerotic lesions, rose by up to 70 times. In mammals, hypercholesterolemia increases the plasma levels of oxidized LDLs and lipid peroxides, in turn facilitating the adhesion of monocytes and lymphocytes to the vascular endothelium [35,36]. Notably, chlorogenic acid can inhibit the copper-induced oxidation of human LDL in vitro [37]. Chang et al. [38] found that chlorogenic acid suppressed the IL-1β-induced expression of adhesion molecules and induced the production of reactive oxygen species. Therefore, the observed suppression of fluorescent cholesterol accumulation in the caudal artery upon dietary supplementation with CPPs may be attributable to the inhibition, by CPP components, of cholesterol or lipoprotein oxidation (or both) and to reduced expression of adhesion molecules of the vascular endothelium.

We showed here that CPPs exert hypocholesterolemic effects and inhibit cholesterol accumulation in blood vessels. However, the effects of coffee ingestion on hypercholesterolemia and cardiovascular disease remain controversial [39,40]. Although coffee is rich in CPPs, coffee contains several other potentially bioactive compounds, including caffeine, vitamin B3, magnesium, potassium, fibrous materials, and hydroxyhydroquinone [40,41]. This last compound, which is a generator of reactive oxygen species, compromises chlorogenic acid–induced improvements in blood pressure and endothelial function [42]. Further study is needed to define the coffee compounds exerting beneficial or detrimental effects on cholesterol metabolism and the vascular endothelial system.

## Conclusions

We showed here that CPP ingestion by hypercholesterolemic zebrafish suppressed cholesterol accumulation in the plasma, liver, and vascular system via downregulation of cholesterol and lipoprotein synthesis and upregulation of the synthesis of bile acids. Our results suggest that CPPs can prevent the development of hypercholesterolemia in humans and that CPP ingestion may help in the management of this condition.

## Abbreviations

CPPs: Coffee polyphenols; CQA: Caffeoylquinic acid; diCQA: Dicaffeoylquinic acid; FQA: Feruloylquinic acid; LDL: Low-density lipoprotein; Cyp7a1: Gene encoding cytochrome P450 polypeptide 1a of subfamily A of family 7(*Mus musculus*); cyp7a1a: Gene encoding cytochrome P450 polypeptide 1a of subfamily A of family 7(*Danio rerio*); eef1a1l1: Gene encoding eukaryotic translation elongation factor 1 alpha 1-like 1 (*Danio rerio*); hmgcra: Gene encoding 3-hydroxy-3-methylglutaryl-coenzyme A reductase A (*Danio rerio*); Hmgcr: Gene encoding 3-hydroxy-3-methylglutaryl-coenzyme A reductase (*Mus musculus*); mtp: Gene encoding microsomal triglyceride transfer protein (*Danio rerio*); Mttp: Gene encoding microsomal triglyceride transfer protein (*Mus musculus*); HMGCR: 3-hydroxy-3-methylglutaryl-coenzyme A reductase (*Mus musculus*).

## Competing interests

No author has any competing interest to declare.

## Authors’ contributions

SM conducted all the animal studies. TH1 performed RNA extractions and quantitative real-time PCR analysis. SM drafted the manuscript with the help of the other authors. All authors were involved in conception of the study and participated in study design and coordination. All authors have read and approved the final manuscript.

## Authors’ information

Shinichi Meguro, PhD, is a senior principal research scientist in R&D – Biological Science Research at Kao Corporation. Dr. Meguro’s research focus is lipid metabolism, nutrition, and health science.

Takahiro Hasumura is a research scientist in R&D – Biological Science Research at Kao Corporation. Mr. Hasumura’s research focus is health science.

Tadashi Hase DVM is a Vice-President with responsibility for R&D – Biological Science Research at Kao Corporation. Dr. Hase’s research focus is lipid metabolism, nutrition, and health science.
